# Meibomian Gland Assessment in Routine Ophthalmology Practice

**DOI:** 10.3390/metabo13020157

**Published:** 2023-01-20

**Authors:** Igor Petriček, Martina Tomić, Tomislav Bulum, Dina Lešin Gaćina, Sania Vidas Pauk

**Affiliations:** 1 Department of Ophthalmology, Zagreb University Hospital Center, 10000 Zagreb, Croatia; 2 School of Medicine, University of Zagreb, 10000 Zagreb, Croatia; 3 Vuk Vrhovac University Clinic for Diabetes, Endocrinology and Metabolic Diseases, Merkur University Hospital, 10000 Zagreb, Croatia

**Keywords:** meibomian gland, dry eye syndrome, meibomian gland dysfunction, meibomian gland lipids, sebaceous glands, sex hormones

## Abstract

This cross-sectional study aimed to investigate the connection between meibomian gland (MG) excreta quantity and quality after MG expression (MGX), dry eye disease (DED) symptoms, and objective DED signs and to clarify the relationship between dry eye and MG function in DED pathophysiology. The study included 200 subjects, 100 with and 100 without dry eye symptoms. Schein questionnaire was used to determine the severity of dry eye symptoms and self-reported skin type for facial skin dryness self-evaluation. Objective dry eye signs were assessed by monitoring conjunctival hyperemia, lid parallel conjunctival folds (LIPCOF), tear break-up time (TBUT), fluorescein surface staining and digital MGX. Subjects with DED symptoms had significantly lower MG quantity scores than healthy controls (*p* < 0.001). Meibum quality and quantity scores significantly correlated with female gender (*p* = 0.002), Schein questionnaire score (*p* < 0.001), fluorescein corneal staining score (*p* = 0.019), self-reported skin type (*p* < 0.001), TBUT (*p* < 0.001) and LIPCOF (*p* = 0.041). After adjustment for age and gender in a logistic regression analysis, dry eye was independently and significantly associated with self-reported skin type (OR 0.73, *p* < 0.001), LIPCOF (OR 1.04, *p* < 0.001), fluorescein corneal staining (OR 1.05, *p* = 0.019), TBUT (OR 0.77, *p* < 0.001) and meibum quantity score (OR 0.59, *p* < 0.001). Dry eye symptoms and objective signs correlated well in this study. MGX discriminated between the subjects with and without DED symptoms and was associated with other objective DED signs. Results showed a significant association between meibum quality and quantity, MG function, DED and facial skin dryness self-perception. This paper established a correlation between dry eye symptoms caused by MG dysfunction and dry skin, which can help general health practitioners consider dry eye as a cause of chronic eye complaints with patients who report dry skin.

## 1. Introduction

In the last three decades, dry eye disease (DED) has been recognized as a growing public-health concern causing several disorders that impair quality of life, such as ocular discomfort, fatigue and visual disturbance. Nowadays, DED is defined as a multifactorial disease from various causes that interact and result in diminished tear film homeostasis, such as age, gender, hormone disbalance and vitamin deficiency, environmental and iatrogenic factors, inflammation, low blink rate, lid disorders and psychological causes [1]. In populations older than 50, DED has a high prevalence, affecting up to 30–50% of the population, even reaching up to 75% in some reports [2]. Women have a 1–1.5-times higher prevalence rate of signs and symptoms of DED than men [2].

Meibomian gland dysfunction (MGD), representing an evaporative component of DED, is recognized as the most common underlying cause of dry eye in population-based and clinical trials [3]. Changes in Meibomian lipid chemistry result in meibomian gland (MG) obstruction, atrophy and quantitative and qualitative changes in the meibum, leading to tear film alteration and eye-irritation symptoms. Obstructive MGD is the most prevalent form of MGD, and it is one of the most underrecognized diseases in ophthalmic practice, representing a chronic abnormality of MG. Usually, it is not accompanied by symptomatic inflammation of the eyelids or obvious signs of the pathology of the eyelid margins, which is why it can often be missed during routine slit lamp examination [4].

MGs secrete tear film meibum, and meibum quantity, quality and expressibility reflect meibomian gland function [5]. It is commonly assessed by the MG expression (MGX). MGX is performed by applying digital pressure to MG along the eyelid skin surface on the eyelid margins [6,7] or using a diagnostic expression instrument [8,9]. When MG function is normal, meibum is clear and readily expressed with gentle pressure. With the loss of clarity in MGD, the meibum can become cloudy, opaque and foamy, and its viscosity might increase, becoming difficult to express and taking on the consistency of toothpaste. [5]. Various grading schemes are developed to evaluate ranging meibum quality, quantity and expressibility. [6,7,10,11,12,13]. Different scoring systems evaluate MG response to varying levels of digital pressure along the glands and assess the number and location of expressible glands, offering valuable information on MG status. The International Workshop on Meibomian Gland Dysfunction subcommittee recommended the mandatory use of MGX in the dry eye diagnostic scheme since obstructive MGD, which occurs without obvious blepharitis and other visible signs of inflammation, can only be accurately detected by the physical expression of MG [4].

Females are at significant risk for developing DED, and DED occurs more frequently in older people. Androgens are the essential hormones in regulating lacrimal, meibomian and sebaceous glands’ function, and imbalance in these hormones results in aqueous-deficient and evaporative DED [14,15,16,17]. Androgens regulate over 1000 genes involved in the normal function of MGs and suppress keratinization, obstruction and atrophy of MGs [17,18,19,20,21,22,23,24,25]. On the other hand, androgen deficiency increases the risk of MGD and evaporative DED [8,11,12,13,14,15]. Androgen deficiency is typically present during menopause in women, in older age in both sexes and in some autoimmune diseases [18,26,27].

Estrogen has an antagonistic action to androgens and upregulates genes that have the opposite effects on lipid production [19,28]. Androgens and estrogens primarily affect sebocytes and the modification of their relative quantity causes a trend change in sebum lipids [19,29]. In addition to sex hormones, some other hormones, such as growth hormone, insulin-like growth factor 1 and hypothalamic–pituitary hormones, modulate and improve the function and growth of MG and sebaceous glands [18,30]. Older age is characterized by androgen deficiency and, consequently, by a reduction in meibum and sebum secretion [30,31].

Meibum is a wax with a melting point of around 25 degrees centigrade. In a cold environment, the eyelid cools down and the meibum becomes solid in the gland ducts and is consequently not secreted on the ocular surface, causing hyperevaporation of the aqueous segment of tears. Therefore, it is not surprising that dry eye symptoms are more frequent and more severe during winter, which stresses the importance of environmental factors, such as relative humidity and outdoor temperature [18,32].

We previously found a connection between dry eye symptoms and facial skin dryness, mainly present in older women [33]. Along with dry eye, dry skin is the most common skin disorder with a high prevalence in individuals over 65 years, and most patients with DED also complain about having dry skin [34,35,36]. Like dry eye, underlying conditions related to dry skin are reduced activity of sebaceous and sweat glands with reduced androgens and estrogen levels. In patients with dry skin, sebaceous glands do not produce enough oily substances called sebum to protect them from losing water, making their skin feel dry.

According to that, this study aimed to further clarify the relationship between dry eye and MGD in DED pathophysiology and its connection to dry skin.

## 2. Materials and Methods

### 2.1. Study Design and Subjects 

This cross-sectional study was performed in a tertiary eye-care center. It was approved by the institutional Ethics Committee of the University Hospital Centre Zagreb following the Helsinki Declaration. The study patients were orally informed about the study protocol and signed written informed consent.

Thus, 200 subjects were selected over a six-month period during the authors’ routine clinical outpatient practice. At the first visit, after signing informed consent, a standardized Schein questionnaire was administered to determine the severity of DED symptoms [37,38,39,40]. The subjects were also asked about their facial skin dryness, which they graded on a scale from 1 (very dry) to 10 (very oily) [35]. Objective dry eye signs were assessed by noting conjunctival hyperemia (CCRLU), lid-parallel conjunctival folds (LIPCOF), fluorescein tear break-up time (TBUT) and fluorescein surface staining, while meibomian gland assessment was performed after MGX. A single examiner, the first author of the present work, performed all the measurements, which were reperformed and rechecked by the fifth author. All the measures that differed between the two observers were discarded. Eligible patients had to be 18 or older, have normal other anterior ocular surface findings, not be contact-lens wearers and not use topical ophthalmic therapy. Patients who had an acute eye infection, previous eye trauma, glaucoma, other ocular surface diseases and irregularities, ocular surgery in the past year, systemic disorders or used drugs (systemic antibiotics, steroid hormones, immunosuppressive drugs, etc.) that might affect the ocular surface and had poor cooperation were not included in the study. 

### 2.2. Schein Questionnaire

This six-item questionnaire was used to assess the extent and severity of dry eye symptoms that patients scored on a scale from 0 to 4 (0—none, 1—rarely, 2—sometimes, 3—often and 4—all the time) [37,38,39,40]. Higher scores indicated more problems or symptoms, ranging from 0 to 24. The questionnaire is commonly used in the authors’ routine clinical work since it is validated and translated into the Croatian language [29,31,35]. A significant advantage is that it is not protected by copyright and is, therefore, widely available without restriction. Further, it is practical, short, simple and understandable to all patients, especially the elderly, so it is frequently used in clinical practice. The questionnaire’s disadvantages are that it has no validated cut-off value for DED and does not evaluate the influence of DED symptoms on everyday activities, vision quality and quality of life (QoL). 

### 2.3. Objective Dry Eye Signs

Conjunctival hyperemia was assessed using the Cornea and Contact Lens Research Unit (CCLRU) grading scale, which was scored from 0 to 4 (0—absent, 1—very slight, 2—slight, 3—moderate and 4—severe) [41].

LIPCOF was assessed in the lower lateral quadrant of the bulbar conjunctiva, parallel to the lower lid margin, without fluorescein, and graded on a scale from 0 to 3 (0—no conjunctival folds, 1—one permanent and clear parallel fold, 2—two permanent and clear parallel folds and 3—more than two permanent and clear parallel folds) [42].

TBUT was measured using standardized fluorescein strips (Biotech, Minneapolis, MN, USA, Fluorescein Sodium Ophthalmic Strip USP). Three consecutive TBUT measurements were performed first on the left eye and then on the right eye [5].

Fluorescein corneal staining was assessed using National Eye Institute/Industry Workshop (NEI) scale [38]. According to that scale, the cornea was divided into five zones: central, upper, lower, nasal and temporal; the staining intensity was graded from 0 (absent) to 3 (severe) in each zone based on the amount, size and confluence of the punctate keratitis. The maximum score is 15.

Meibomian gland assessment was performed after MGX on the lower eyelid of both eyes after other dry eye tests to obtain them unaffected by MGX. Korb’s description of the digital expression of Meibomian glands was used [43]. Meibum quantity (MG expressibility) was assessed according to the scale: 3—most MG ducts secrete a clear meibum under-expression; 2—about half of the excretory MG ducts secrete meibum, the secretion is scarce; 1—a few ducts secrete meibum, barely visible secretion; 0—there is no visible MG secretion after the expression. Meibum quality was assessed according to the scale: 4—clear; 3—cloudy; 2—cloudy with debris (granular); 1—toothpaste-like.

### 2.4. Statistical Analysis

Statistical analysis was performed in the Statistica software package version 14.0 (TIBCO Inc., Palo Alto, CA, USA) and SPSS software package version 23.0. (IBM, Armonk, NY, USA). After testing the normality of data distribution via the Kolmogorov–Smirnov test, the appropriate parametric and nonparametric tests were used. For continuous and ordinal data, differences between two groups were tested by *t*-test or Mann–Whitney test for independent variables, while the Wilcoxon test was used for dependent variables. For categorical data testing, the Chi-square test was used. Continuous data were presented as mean ± SD and median (min–max), ordinal data as median (min–max) and categorical data as numbers. The Spearman rank correlation test evaluated the presence of associations between examined variables, and binary univariate and multiple logistic regression analyses were performed to determine the strength and independence of associations. In all analyses, the level of statistical significance was set at 0.05.

## 3. Results

### 3.1. Baseline Characteristics and Skin Type 

Two hundred subjects (45 males/155 females, mean age 46.28 ± 16.34 years) were included in this study and, according to the dry eye symptoms assessed by the Schein questionnaire, divided into two groups: dry eye group (*n* = 100)—subjects with dry eye symptoms (Schein questionnaire score 1–24), and control group (*n* = 100)—subjects without symptoms (Schein questionnaire score 0). 

The baseline characteristics and skin type of subjects included in the study are presented in Table 1. Subjects with dry eye symptoms were significantly older than control subjects (*p* < 0.001), and there were significantly more women in the dry eye group than in the control group (*p* < 0.001). The median Schein questionnaire score in the dry eye group was 7 (min 1–max 24), whereas, in the control group, it was 0. Further, subjects with dry eye symptoms self-reported drier facial skin than those without these symptoms (*p* < 0.001).

### 3.2. Objective Dry Eye Signs and Meibomian Gland Function

Since there were no significant differences, using the Wilcoxon test, in the objective dry eye sign scores as well as in meibum quantity and quality scores after MGX between the right and left eye within the examined groups in the further statistical analyses, the mean values of both eyes (median, min-max) were used. Table 2 presents the objective dry eye sign scores and meibum quantity and quality scores after MGX of both eyes in subjects divided into two groups according to the presence or absence of dry eye symptoms. The two groups did not significantly differ in conjunctival hyperemia (CCRLU) (*p* = 0.626). LIPCOF was higher and TBUT shorter in dry eye subjects than in subjects without dry eye symptoms (*p* < 0.001). The fluorescein corneal staining score of both eyes was higher in dry eye subjects than in the control group (*p* = 0.047). The meibum quantity score after MGX of both eyes was significantly lower in the dry eye group than in the control group (*p* < 0.001), while no significant difference among groups was observed in the meibum quality scores after MGX of both eyes (*p* = 0.205).

### 3.3. Correlation between Meibomian Gland Function, Baseline Characteristics, Skin Type, Schein Questionnaire and Objective Dry Eye Signs

**Figure 1:**
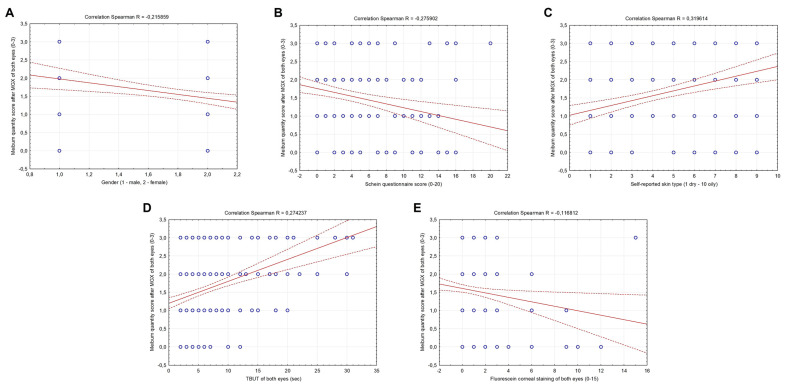
Correlations between the meibum quantity score after MGX, gender (**A**), Schein questionnaire score (**B**), skin type (**C**), TBUT (**D**) and fluorescein corneal staining (**E**) of both eyes in all subjects included in the study.

Meibum quantity score after MGX of both eyes was significantly correlated with the female gender (R = −0.215859, *p* = 0.002), negatively with Schein questionnaire score (R = −0.275902, *p* < 0.001) and fluorescein corneal staining score of both eyes (R = −0.116812, *p* = 0.019), whereas significantly positively with self-reported skin type (1 dry–10 oily) (R = 0.319614, *p* < 0.001) and TBUT score of both eyes (R = 0.274237, *p* < 0.001) (Figure 1A–E). No significant correlation was observed between the meibum quantity score and age, the CCRLU and the LIPCOF score of both eyes (*p* > 0.05) (data not shown).

**Figure 2:**
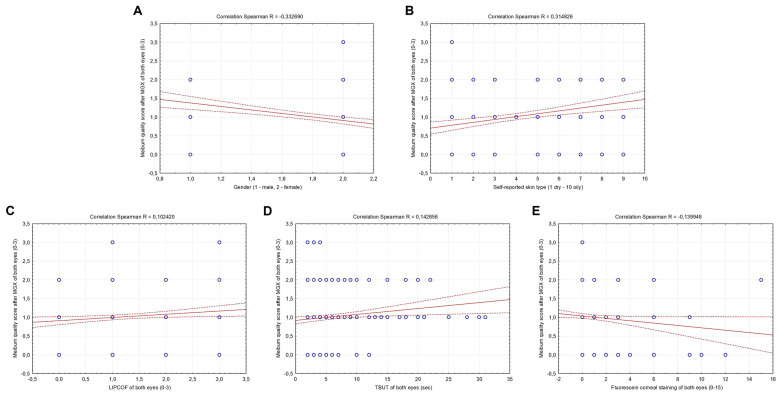
Correlations between the meibum quality score after MGX, gender (**A**), skin type (**B**), LIPCOF (**C**), TBUT (**D**) and fluorescein corneal staining (**E**) of both eyes in all subjects included in the study.

Meibum quality score after MGX of both eyes was also significantly correlated with the female gender (R = −0.332690, *p* < 0.001), negatively with fluorescein corneal staining score of both eyes (R = −0.139948, *p* = 0.005), whereas significantly positively with self-reported skin type (1 dry–10 oily) (R = 0.314826, *p* < 0.001), LIPCOF (R = 0.102420, *p* = 0.041) and TBUT score of both eyes (R = 0.142656, *p* = 0.004) (Figure 2A–E). No significant correlation was observed between the meibum quality score A–E and age, the Schein questionnaire score and the CCRLU score of both eyes (*p* > 0.05) (data not shown).

### 3.4. Predictors and Indicators of Dry Eye

In the binary univariate logistic regression analysis, the strongest statistically significant associations were found between dry eye and older age (OR 1.64) as well as between dry eye and female gender (OR 4.85), with a parameter estimate of 0.03908 (*p* < 0.001) and 1.57818 (*p* < 0.001) relative to a one-unit change in age and gender (Table 3). Furthermore, other factors associated with an increased risk of dry eye were the presence of dry skin (OR 0.73, *p* < 0.001), higher LIPCOF (OR 1.04, *p* < 0.001), higher fluorescein corneal staining score (OR 1.05, *p* = 0.019), shorter TBUT (OR 0.77, *p* < 0.001) and lower meibum quantity score (OR 0.59, *p* < 0.001). However, when standardized for age and gender, in multiple regression analysis, all mentioned factors were independently and significantly associated with dry eye (*p* < 0.05).

## 4. Discussion

Dry eye symptoms and signs usually have a weak correlation, while diagnostic tests for DED present considerable inconsistency [44]. Therefore, a combination of various questionnaires and clinical examinations are used to diagnose dry eye, as none of them alone would adequately detect patients with DED. The high and rapidly increasing incidence of DED pushes forward the need to find simple and affordable diagnostic approaches and techniques that could be used in daily practice, and treatment is best individualized by targeting specific mechanisms that trigger dry eye symptoms in each patient. Therefore, since it is the main and the most common etiologic factor in the pathogenesis of DED, obstructive MGD should be appropriately diagnosed and treated. The present study’s authors wanted to investigate the correlation between MG function assessed by the quantity and quality of the meibum evaluated after digital MGX, dry eye symptoms and other objective DED signs. Further, they wanted to determine the clinical applicability, utility and objectivity of digital MGX in assessing MG for use in routine clinical practice. 

In the present study, subjects who reported dry eye symptoms had significantly lower meibum quantity scores after MGX than controls. It pointed out that, in this way, performing digital MGX might discriminate between patients with symptoms of DED and healthy subjects. In a study published by Pfugfelder and coauthors, digital MGX was used to obtain MG function in patients with MGD and aqueous tear deficiency [11]. The study revealed that subjects with both types of DED had lower MG expressibility than healthy subjects; also, patients with MGD had the lowest MGX scores. Shimazaki et al. reported some degree of MGD obstruction assessed by digital MGX in patients with aqueous tear deficiency that was more severe in patients with Sjogren syndrome [12]. On the other hand, Mathers et al., using digital pressure MGX in blepharitis and healthy controls, found an increased volume of MG excreta with increased viscosity [6]. Korb and Henriquez found that during the expression of MG under mild and forceful digital pressure, the glands of the lower eyelid in asymptomatic individuals secreted at a significantly higher frequency than in patients with dry eye [45]. Korb and Blackie developed a custom-made device that performed standardized force on the lower lid (1.25 g/mm^2^ for 10–15 s), which allowed for the enumeration of liquid meibum-expressible glands (Meibomian Glands Yielding Liquid Secretion (MGYLS)) [8]. A negative correlation was found between the severity of DED symptoms and the number of MGYLSs. Similarly, Finis et al. found a significant correlation between MG expressibility obtained by a small handheld instrument (TearScience, Inc.) and lipid layer thickness assessed by a LipiView interferometer (TearScience, Inc.) [9]. As is seen, MGX was described by many authors; however, its role in DED still needs to be established. The need for more consensus on performing a standardized MGX technique and grading the MG excreta hampered its broad adoption in clinical practice. However, the results of different studies, despite being hardly comparable due to the various methodologies and diagnostic schemes used, highlighted the significant correlation between abnormal MG secretion, MGD and dry eye and pointed out the importance of using it in DED diagnostics.

The current study presented a good correlation between dry eye symptoms and objective signs investigated therein. The meibum quantity and quality scores significantly correlated with the female gender, the common risk factor for developing MGD due to hormonal specificities in menopausal women and aging. Both meibum quantity and quality after digital MG expression were significantly related to dry eye symptoms, fluorescein corneal staining score, TBUT and LIPCOF. These findings again confirmed a significant relationship and association between MGD and DED. Further, they established the objectivity of the currently described method in the assessment and detection of MGD. 

One of the additional observations in this study was a comparison between DED signs and symptoms and skin dryness. The meibum quantity and quality scores positively correlated with self-reported skin type. The subjects, mostly women, who complained more frequently of dry skin, had lower meibum quality and quantity scores. In their previous works, the authors of the present study found a significant correlation between dry skin and dry eye [29,30,35]. They proposed that the relation might be on the hormonal level. MG and sebaceous glands have shared hormones and the same etiological hormonal pathways might induce down-regulation, as described in more detail in the Introduction. Therefore, the subjects who complain of skin dryness due to dysfunction of sebaceous glands (SGs) might simultaneously suffer from ocular dryness caused by MGD. Recognition and awareness of these symptoms can be critical to the early and timely detection of both conditions. Moreover, since similar pathophysiological pathways are proposed, it might help to find new therapeutic approaches for both states in the future.

Logistic regression analysis presented lower meibum quantity scores, higher TBUT, LIPCOF, fluorescein surface staining and self-reported dryer skin as strong indicators and predictors of dry eye complaints. 

Therefore, in this study, MGX performed by digital pressure of MG, as described in the Methods, showed a good correlation with dry eye symptoms and other objective dry eye signs. The results showed meibum quality score to be a good indicator and predictor of dry eye symptoms. The method is presented as accurate and objective, which, in the first place, uses a simple and accessible methodology. Almost every eye specialist can use it for routine dry eye diagnostics in daily ophthalmology practice. The results of this study also highlighted a significant correlation between pathological MG secretion and DED, as well as a significant correlation between altered MG secretion and self-reported dry skin. That may indicate the connection between MGD and sebaceous gland dysfunction. It is essential to investigate it in further studies since one condition may come together with another and might help diagnose both disorders and explore new therapeutic approaches. 

The authors had to address the potential limitations of this study. Using the Schein questionnaire represented a potentially significant burden. The most important limitation of the questionnaire was that it does not have a validated cutoff value for dry eye, regardless of its scored responses. This could have influenced the classification of the patients into the study groups. Further, it does not evaluate the influence of DED on everyday activities, vision quality and QOL, while some other questionnaires do, e.g., the 5-Item Dry Eye Questionnaire (DEQ-5) and Ocular Surface Disease Index (OSDI). However, the authors chose it because they commonly use it in clinical practice, have experience with its implementation and, most importantly, it is not protected by copyright.

Secondly, there are no references in the literature on how to diagnose dry skin, which diagnostic tests, signs, symptoms or questionnaires use for accurate assessment of dry skin because it is still not recognized as a disease, and its pathophysiology is unclear and needs to be investigated in more detail. In the present work, the authors performed a self-reported facial skin query, which was used similarly in other studies [29,30,35,46,47].

Another potential limitation is that the authors did not use other newly developed methods for MG assessment, such as meibography using LacryDiag (Quantel Medical) or LipiView (TearScience, Inc.). Unfortunately, these instruments were unavailable in their routine work and are not available to the vast majority of eye care practitioners. Therefore, one of the essential goals of this study was an assessment of a simple and readily available MGD diagnostic procedure that virtually any eye care practitioner can use in their everyday clinical practice. 

In the pathophysiology of MGD, hormonal imbalance between androgens and estrogens plays an important etiological role. It is, therefore, clear that the eye and DED should not be viewed as just an eye condition but as a general body surface problem and a potential endocrinological problem. This raises the question of whether MGD and dry eye may indicate other endocrinological changes and conditions.

## 5. Conclusions

This study confirmed a significant correlation between MG secretion, MG function and DED. Digital MGX was presented as an objective method that discriminated between subjects who complained of DED symptoms and healthy controls and correlated well with other objective DED signs. Further, it was presented as a good indicator and predictor of dry eye complaints. In the present study, subjects who self-perceived their facial skin as dryer had pathological MG secretion, bringing into focus the connection between MG and SG function, which is already proposed that it might be on the level of the sex hormones and other hormonal pathways. It is essential to investigate this more profoundly, at the genetic and molecular levels, since these findings could help to find new diagnostic and therapeutic approaches in dry eye management.

The data presented here might help general health practitioners as well. Patients complaining of chronic eye symptoms who have dry skin might also have dry eye as the underlying ocular condition; therefore, this information might help choose optimal diagnostic and therapeutic options.

## Figures and Tables

**Table 1:** Baseline characteristics and skin type of subjects included in the study.

	Dry Eye Group (*n* = 100)	Control Group (*n* = 100)	t ^a^ Chi ^b^	*p*
Age (years) *	51.17 ± 15.16	41.39 ± 16.08	4.426 ^a^	<0.001 ^a^
Gender (m/f) **	10/90	35/65	17.921 ^b^	<0.001 ^b^
Schein questionnaire score *	7 (1–20)	0 (0–0)	12.216 ^c^	<0.001 ^c^
Self-reported skin type (1 dry–10 oily) †	2 (1–9)	5 (1–9)	−5.049 ^c^	<0.001 ^c^

* $\bar{x}$ ± SD, ** numbers, † Med (Min–Max), ^a^ *t*-test, df = 198, ^b^ Chi-square test, df = 1, ^c^ Mann–Whitney test.

**Table 2:** The objective dry eye signs and meibum quantity and quality scores after MGX of both eyes in subjects divided into two groups according to the presence/absence of dry eye symptoms.

	Dry Eye Group (*n* = 100)	Control Group (*n* = 100)	Z	*p*
CCRLU of both eyes (0–4)	0 (0–2)	0 (0–0)	0.487	0.626
LIPCOF of both eyes (0–3)	1.5 (0–3)	1 (0–3)	5.142	<0.001
TBUT of both eyes (s)	4 (2–12)	5 (2–28)	−5.035	<0.001
Fluorescein corneal staining of both eyes (0–15)	0 (0–15)	0 (0–3)	1.980	0.047
Meibum quantity score of both eyes (0–3)	1 (0–3)	2 (0–3)	−3.532	<0.001
Meibum quality score of both eyes (0–3)	1 (0–3)	1 (0–2)	−1.259	0.208

Med (Min–Max), Mann–Whitney test. Abbreviations: CCRLU: cornea and contact lens research unit; LIPCOF: lid-parallel conjunctival folds; TBUT: tear break-up time.

**Table 3:** Predictors and indicators of dry eye symptoms using binary logistic regression analysis.

	Parameter Estimate	Wald’s Chi-Square	*p*	OR (95%CI)	Parameter Estimate	Wald’s Chi-Square	*p*	AOR (95% CI) *
Age (years)	0.03908	16.956	<0.001	1.64 (1.09–2.48)	/			
Gender (female)	1.57818	16.056	<0.001	4.85 (2.23–9.94)	/			
Self-reported drier or oilier facial skin (1–10)	−0.31468	20.799	<0.001	0.73 (0.64–0.84)	−0.1732	4.886	0.027	0.84 (0.72–0.98)
LIPCOF of both eyes	1.13481	26.879	<0.001	1.04 (1.02–1.06)	0.77804	10.022	0.002	1.18 (1.04–1.54)
TBUT of the right eye	−0.26232	20.889	<0.001	0.77 (0.69–0.86)	−0.23057	15.263	<0.001	0.79 (0.71–0.89)
Fluorescein corneal staining of both eyes	0.43914	5.537	0.019	1.05 (1.01–1.24)	0.49599	5.617	0.028	1.14 (1.09–1.48)
Meibum quantity score of both eyes	−0.53517	12.957	<0.001	0.59 (0.44–0.79)	−0.42891	7.044	0.008	0.65 (0.47–0.89)
Omnibus tests χ^2^ = 84.840, df = 7, *p* < 0.001 Cox & Snell R^2^ = 0.346, Nagelkerke R^2^ = 0.461 Hosmer and Lemeshow test χ^2^ = 5.354, df = 8, *p* = 0.719					Omnibus tests χ^2^ = 77.145, df = 5, *p* < 0.001 Cox & Snell R^2^ = 0.320, Nagelkerke R^2^ = 0.427 Hosmer and Lemeshow test χ^2^ = 7.069, df = 8, *p* = 0.529			

* OR standardized for age and gender. Abbreviations: CCRLU: cornea and contact lens research unit; LIPCOF: lid-parallel conjunctival folds; TBUT: tear break-up time; MGX: meibomian gland expression.

## Data Availability

The authors confirm that the data supporting the findings of this study are available within the article, and from the corresponding author upon request.

## References

[1] Bron A.J., de Paiva G.S., Chauhan S.K., Bonini S., Gabison E.E., Jain S., Knop E., Markoulli M., Ogawa Y., Perez V. (2017). TFOS DEWS II pathophysiology report. Ocul. Surf..

[2] Stapleton F., Alves M., Bunya V.Y., Jalbert I., Lekhanont K., Malet F., Na K.S., Schaumberg D., Uchino M., Vehof J. (2017). TFOS DEWS II Epidemiology Report. Ocul. Surf..

[3] Craig J.P., Nicholsm K.K., Akpek E.K., Caffery B., Dua H.S., Joo C.K., Liu Z., Nelson J.D., Nichols J.J., Tsubota K. (2017). TFOS DEWS II Definition and Classification Report. Ocul. Surf..

[4] Tomlinson A., Bron A.J., Korb D.R., Amano S., Paugh J.R., Pearce E.I., Yee R., Yokoi N., Arita R., Dogru M. (2011). The international workshop on meibomian gland dysfunction: Report of the diagnosis subcommittee. Investig. Ophthalmol. Vis. Sci..

[5] Wolffsohn J.S., Arita R., Chalmers R., Djalilian A., Dogru M., Dumbleton K., Gupta P.K., Karpecki P., Lazreg S., Pult H. (2017). TFOS DEWS II Diagnostic Methodology report. Ocul. Surf..

[6] Mathers W.D., Shields W.J., Sachdev M.S., Petroll W.M., Jester J.V. (1991). Meibomian gland dysfunction in chronic blepharitis. Cornea.

[7] Bron A.J., Benjamin L., Snibson G.R. (1991). Meibomian gland disease. Classification and grading of lid changes. Eye.

[8] Korb D.R., Blackie C.A. (2008). Meibomian gland diagnostic expressibility: Correlation with dry eye symptoms and gland location. Cornea.

[9] Finis D., Pischel N., Schrader S., Geerling G. (2013). Evaluation of lipid layer thickness measurement of the tear film as a diagnostic tool for Meibomian gland dysfunction. Cornea.

[10] Mathers W.D., Shields W.J., Sachdev M.S., Petroll W.M., Jester J.V. (1991). Meibomian gland morphology and tear osmolarity: Changes with Accutane therapy. Cornea.

[11] Pflugfelder S.C., Tseng S., Sanabria O., Kell H., Garcia C.G., Felix C., Reis B.L. (1998). Evaluation of subjective assessments and objective diagnostic tests for diagnosing tear-film disorders known to cause ocular irritation. Cornea.

[12] Shimazaki J., Goto E., Ono M., Shimmura S., Tsubota K. (1998). Meibomian gland dysfunction in patients with Sjögren syndrome. Ophthalmology.

[13] Henriquez A.S., Korb D.R. (1981). Meibomian glands and contact lens wear. Br. J. Ophthalmol..

[14] Azcarate P.M., Venincasa V.D., Feuer W., Stanczyk F., Schally A.V., Galor A. (2014). Androgen deficiency and dry eye syndrome in the aging male. Investig. Ophthalmol. Vis. Sci..

[15] Truong S., Cole N., Stapleton F., Golebiowski B. (2014). Sex hormones and the dry eye. Clin. Exp. Optom..

[16] Gagliano C., Caruso S., Napolitano G., Malaguarnera G., Cicinelli M.V., Amato R., Reibaldi M., Incarbone G., Bucolo C., Drago F. (2014). Low levels of 17-β-oestradiol, oestrone and testosterone correlate with severe evaporative dysfunctional tear syndrome in postmenopausal women: A case–control study. Br. J. Ophthalmol..

[17] Takayasu S., Wakimoto H., Itami S., Sano S. (1980). Activity of testosterone 5 alpha-reductase in various tissues of human skin. J. Investig. Dermatol..

[18] Sullivan D.A., Jensen R.V., Suzuki T., Richards S.M. (2009). Do sex steroids exert sex-specific and/or opposite effects on gene expression in lacrimal and meibomian glands?. Mol. Vis..

[19] Steagall R.J., Yamagami H., Wickham L.A., Sullivan D.A. (2002). Androgen control of gene expression in the rabbit meibomian gland. Adv. Exp. Med. Biol..

[20] Yamagami H., Schirra F., Liu M., Richards S.M., Sullivan B.D., Sullivan D.A. (2002). Androgen influence on gene expression in the meibomian gland. Adv. Exp. Med. Biol..

[21] Schirra F., Richards S.M., Liu M., Suzuki T., Yamagami H., Sullivan D.A. (2006). Androgen regulation of lipogenic pathways in the mouse meibomian gland. Exp. Eye Res..

[22] Schirra F., Richards S.M., Sullivan D.A. (2007). Androgen influence on cholesterogenic enzyme mRNA levels in the mouse meibomian gland. Curr. Eye Res..

[23] Sullivan B.D., Evans J.E., Cermak J.M., Krenzer K.L., Dana M.R., Sullivan D.A. (2002). Complete androgen insensitivity syndrome: Effect on human meibomian gland secretions. Arch. Ophthalmol..

[24] Yamagami H., Richards S.M., Sullivan B.D., Liu M., Steagall R.J., Sullivan D.A. (2002). Gender-associated differences in gene expression of the meibomian gland. Adv. Exp. Med. Biol..

[25] Labrie F., Bélanger A., Cusan L., Gomez J.L., Candas B. (1997). Marked decline in serum concentrations of adrenal C19 sex steroid precursors and conjugated androgen metabolites during aging. J. Clin. Endocrinol. Metab..

[26] Versura P., Campos E.C. (2005). Menopause and dry eye. A possible relationship. Gynecol. Endocrinol..

[27] Suzuki T., Schirra F., Richards S.M., Jensen R.V., Sullivan D.A. (2008). Estrogen and Progesterone Control of Gene Expression in the Mouse Meibomian Gland. Investig. Ophthalmol. Vis. Sci..

[28] Chen F., Hu X., He Y., Huang D. (2021). Lipidomics demonstrates the association of sex hormones with sebum. J. Cosmet. Dermatol..

[29] Petriček I. (2011). The Influence of Tear Film on Visual Function. Ph.D. Thesis.

[30] Clayton R.W., Langan E.A., Ansell D.M., de Vos I.J.H.M., Göbel K., Schneider M.R., Picardo M., Lim X., van Steensel M.A.M., Paus R. (2020). Neuroendocrinology and Neurobiology of Sebaceous Glands. Biol. Rev. Camb. Philos. Soc..

[31] Vidas Pauk S. (2019). Non-Invasive Tear Break-Up Time Measurement Using Handheld Lipid Layer Thickness Assessment Tool. Ph.D. Thesis.

[32] Sunwoo Y., Chou C., Takeshita J., Murakami M., Tochihara Y. (2006). Physiological and subjective responses to low relative humidity. J. Physiol. Anthropol..

[33] Petriček I., Pauk S.V., Tomić M., Bulum T. (2021). Dry eye and dry skin—Is there a connection?. Ophthalmic Epidemiol..

[34] Hahnel E., Lichterfeld A., Blume-Peytavi U., Kottner J. (2017). The epidemiology of skin conditions in the aged: A systematic review. J. Tissue Viability.

[35] Moniaga C.S., Tominaga M., Takamori K. (2020). Mechanisms and Management of Itch in Dry Skin. Acta Derm. Venereol..

[36] Guliani B.P., Bhalla A., Naik M.P. (2018). Association of the severity of meibomian gland dysfunction with dyslipidemia in Indian population. Indian J. Ophthalmol..

[37] Schein O.D., Tielsch J.M., Munõz B., Bandeen-Roche K., West S. (1997). Relation between signs and symptoms of dry eye in the elderly. A population-based perspective. Ophthalmology.

[38] Lemp M.A., Foulks G.N. (2007). The definition and classification of dry eye disease: Report of definition and classification subcommittee of international dry eye workshop. Ocul. Surf..

[39] Bakija I., Filipčić I., Bogadi M., Šimunović Filipčić I., Gotovac M., Kaštelan S. (2021). Comparison of the Schein and Osdi Questionnaire as Indicator of Tear Film Stability in Patients with Schizophrenia. Psychiatr. Danub..

[40] Bandeen-Roche K., Muñoz B., Tielsch J.M., West S.K., Schein O.D. (1997). Self-reported assessment of dry eye in a population-based setting. Investig. Ophthalmol. Vis. Sci..

[41] Terry R.L., Schnider C.M., Holden B.A., Cornish R., Grant T., Sweeney D., La Hood D., Back A. (1993). CCLRU standards for success of daily and extended wear contact lenses. Optom. Vis. Sci..

[42] Höh H., Schirra F., Kienecker C., Ruprecht K.W. (1995). Lid-parallel conjunctival folds are a sure diagnostic sign of dry eye. Ophthalmologe.

[43] Korb D.R. (2002). The tear film—Its role today and in the future. The Tear Film, Structure, Function and Clinical Examination.

[44] Thulasi P., Djalilian A.R. (2017). Update in current diagnostics and therapeutics of dry eye disease. Ophthalmology.

[45] Korb D.R., Henriquez A.S. (1980). Meibomian gland dysfunction and contact lens intolerance. J. Am. Optom. Assoc..

[46] Ito K., Takamatsu K., Nohno K., Sugano A., Funayama S., Katsura K., Kaneko N., Ogawa M., Meurman J.H., Inoue M. (2017). Factors associated with mucosal dryness in multiple regions and skin: A web-based study in women. J. Obstet. Gynaecol. Res..

[47] Werschler W.P., Trookman N.S., Rizer R.L., Ho E.T., Mehta R. (2011). Enhanced efficacy of a facial hydrating serum in subjects with normal or self-perceived dry skin. J. Clin. Aesthetic Dermatol..

